# Comparing comorbidity measures for predicting mortality and hospitalization in three population-based cohorts

**DOI:** 10.1186/1472-6963-11-146

**Published:** 2011-06-10

**Authors:** Jacqueline M Quail, Lisa M Lix, Beliz Acan Osman, Gary F Teare

**Affiliations:** 1 Saskatchewan Health Quality Council, Saskatoon, Canada; 2 School of Public Health, University of Saskatchewan, Saskatoon, Canada

## Abstract

**Background:**

Multiple comorbidity measures have been developed for risk-adjustment in studies using administrative data, but it is unclear which measure is optimal for specific outcomes and if the measures are equally valid in different populations. This research examined the predictive performance of five comorbidity measures in three population-based cohorts.

**Methods:**

Administrative data from the province of Saskatchewan, Canada, were used to create the cohorts. The general population cohort included all Saskatchewan residents 20+ years, the diabetes cohort included individuals 20+ years with a diabetes diagnosis in hospital and/or physician data, and the osteoporosis cohort included individuals 50+ years with diagnosed or treated osteoporosis. Five comorbidity measures based on health services utilization, number of different diagnoses, and prescription drugs over one year were defined. Predictive performance was assessed for death and hospitalization outcomes using measures of discrimination (*c*-statistic) and calibration (Brier score) for multiple logistic regression models.

**Results:**

The comorbidity measures with optimal performance were the same in the general population (*n *= 662,423), diabetes (*n *= 41,925), and osteoporosis (*n *= 28,068) cohorts. For mortality, the Elixhauser index resulted in the highest *c*-statistic and lowest Brier score, followed by the Charlson index. For hospitalization, the number of diagnoses had the best predictive performance. Consistent results were obtained when we restricted attention to the population 65+ years in each cohort.

**Conclusions:**

The optimal comorbidity measure depends on the health outcome and not on the disease characteristics of the study population.

## Background

Population-based administrative databases are commonly used in studies about health status and health service utilization. These databases enable easy access to demographic and health-related data on large study populations but they are not without limitations. Studies that use administrative data employ observational designs, which can result in differences amongst study groups, which may also be related to the outcome of interest. This may lead to spurious results if these differences are not controlled using appropriate risk-adjustment methodologies. One of the most important predictors of health-related outcomes is the presence of comorbidities, or pre-existing health conditions that coexist with an index disease [1]. Therefore, for all research related to health-related events and services, it is essential to risk-adjust for comorbidity in order to get unbiased estimates.

A number of comorbidity measures have been applied to administrative data. Some are simple, such as counts of the number of physician visits, diagnoses, or prescription drug dispensations within a prescribed time frame [2]. Comorbidity indices based on specific sets of diagnoses for chronic conditions or prescription drugs used to treat chronic conditions have also been developed. The Chronic Disease Score (CDS) is a weighted index of the burden of comorbid conditions based upon pharmaceutical data from administrative databases [3]. Diagnosis-related indices include the Charlson and Elixhauser indices, which use International Classification of Disease (ICD) diagnosis codes to identify major health problems, such as heart or lung disease [4,5]. Both the Charlson and Elixhauser indices were originally developed using in-hospital populations to predict mortality, although they have been applied to outpatient populations [6-8]. All of these measures are potentially useful for comparing different populations because they are general measures of comorbidity.

Previous research has focused on the performance of these comorbidity measures in either specific populations or for specific outcomes, but never in multiple populations for multiple outcomes. The majority of research has focused on in-patient populations [9-11] and chronic disease populations, including osteoarthritis [6], hypertension [12], and migraine [13]. Most of these studies investigate death or health care costs as the outcome and results have been inconsistent, likely as the result of differences between studies in data sources and how both comorbidity measures and outcomes are defined. More limited research has been conducted in general populations and the research that has been done has focused on mortality as the primary health outcome measure [7,14]. Fewer have investigated health service utilization outcome measures [7,15]. Overall, the majority of studies focus on a single population or outcome and the consistency of the findings across populations and outcomes bears further investigation. With this in mind, we investigated the performance of five comorbidity measures for predicting death and health services utilization outcomes in three population-based cohorts; a general population cohort and two chronic disease cohorts composed of individuals diagnosed with diabetes or osteoporosis.

## Methods

### Data sources

Administrative health data for this research were obtained from the province of Saskatchewan, Canada which has a population of approximately 1.1 million [16]. Data on hospital contacts, physician contacts, and outpatient prescriptions are collected and captured in electronic databases that can be anonymously linked via a unique personal health insurance number [17,18]. Like all Canadian provinces, Saskatchewan has a provincial health insurance plan that virtually all members of the population are registered in except for a relatively small proportion of the population (<1%) whose health care is covered by the federal government (Royal Canadian Mounted Police, veterans, and inmates in federal penitentiaries). Additionally, First Nations people that have treaty relationships with the federal government (approximately 9% of the provincial population) also receive some of their health benefits, including prescription drug benefits, from the federal government. Therefore these individuals are not included in the provincial prescription drug benefit plan and related administrative data files that were used in this study.

Hospital data is stored in the *Discharge Abstract Database*. Diagnoses in hospital data are recorded using the International Classification of Diseases, 9th Revision (i.e., ICD-9) up to and including fiscal year 2001/02, where a fiscal year extends from April 1 to March 31. In fiscal year 2001/02, the International Classification of Diseases, 10th revision, Canadian Version (i.e., ICD-10-CA) was introduced and virtually all codes were recorded in this format from fiscal year 2002/03 onward. Between three and sixteen diagnoses are captured in each record prior to the introduction of ICD-10-CA, and up to 25 diagnoses are captured subsequently. The type of diagnosis is also recorded, which identifies the most responsible diagnosis for admission, comorbid diagnoses that are not directly related to admission, and diagnoses that developed after admission and which represent the development of complications during the hospitalization.

Data on physician services are contained in the *Medical Services Database*. Physicians who are paid on a fee-for-service basis submit billing claims to the provincial health ministry. A single diagnosis using three-digit ICD-9 codes is recorded on each claim. Physicians who are salaried are required to submit billing claims for administrative purposes, a practice known as shadow billing.

The *Prescription Drug Database *contains information on all outpatient drugs dispensed to Saskatchewan residents who are eligible for coverage. Approximately 9% of Saskatchewan residents - primarily Registered Indians - are not eligible because they have their prescription costs paid for by another government agency [18,19]. The database includes information on active ingredients, strength and dosage form, date and quantity dispensed, as well as the pharmacologic-therapeutic classification of a drug based on the American Hospital Formulary System (AHFS) [20].

The *Population Registry *captures demographic characteristics, location of residence, and dates of coverage by the provincial health insurance plan. The *Vital Statistics Registry *contains information on all births and deaths in the province.

The accuracy and completeness of Saskatchewan's administrative databases have made them popular data sources for numerous studies of population health and health services utilization [21-23]. Ethical approval for this research was received from the *University of Saskatchewan Biomedical Research Ethics Board*.

### Cohort definitions

We used these administrative databases to define three cohorts - a general population cohort and two chronic disease cohorts that are subsets of the general population cohort - to investigate whether the performance of comorbidity measures varies among different study populations and by outcome. In order to be included in one of these cohorts, individuals must have had uninterrupted health coverage in the year we assessed comorbidity (fiscal year 2001/02) and the year we assessed outcomes (fiscal year 2002/03). For the diabetes and osteoporosis cohorts, we used data from April 1, 1996 to March 31, 2002 to identify people with either of these conditions.

The general population cohort was composed of all Saskatchewan residents aged 20 and older. We defined the diabetes cohort using the National Diabetes Surveillance System case definition [24], which has been developed using Canadian administrative data and validated in previous research [25,26]. This case definition has been shown to have excellent sensitivity (86%) and specificity (97%) [25]. It identifies all individuals who have a diagnosis of diabetes (ICD-9: 250; ICD-10-CA: E10-E14) in at least one hospital record or in at least two physician claims within a two-year period. The index date is the earliest date of a diabetes diagnosis. We identified all Saskatchewan residents 20 years of age and older who met the case definition using data from April 1, 1996 to March 31, 2002.

We defined the osteoporosis cohort based upon the results of a validation study that evaluated the sensitivity and specificity of osteoporosis diagnosis codes in hospital and physician data, and outpatient prescription drug records for an osteoprotective drug by comparing them to bone mineral densitometry tests from a provincial screening program [15]. The osteoporosis case definition identifies all individuals aged 50 or older who have a diagnosis of osteoporosis (ICD-9: 733; ICD-10-CA: M80, M81) in at least one hospital record or at least one physician claim, or who have at least one outpatient drug dispensation for an osteoprotective medication (i.e., alendronate, clodronate, etidronate, pamidronate, risedronate, salmon calcitonin, raloxifene, teriparatide, zoledronic acid). The case definition has been shown to have a sensitivity of 89.4% and a specificity of 91.5% in women 50 years of age and older [27]. We assigned the index date as the earliest date of diagnosis or prescription drug dispensation. We identified all Saskatchewan residents 50 years of age and older who met the case definition using data from April 1, 1996 to March 31, 2002. Individuals were excluded if they had a diagnosis for Paget's disease (ICD-9: 731.0; ICD-10-CA: M88.0, M88.8, M88.9) in the study period because they may have different comorbidity characteristics than those without the disease.

### Comorbidity measures

Five comorbidity measures were considered: number of different diagnoses, Charlson index, Elixhauser index, number of different dispensed drugs, and the Chronic Disease Score (CDS). Each measure was created using data for fiscal year 2001/02.

The number of different diagnoses recorded to the third digit in ICD-9 and ICD-10-CA was determined using both the hospital and physician billing databases. Any diagnoses related to pregnancy, childbirth, or abortion were excluded because these events are not disease-related.

The Charlson index is a weighted index of the burden of comorbidity used to predict one-year mortality [4]. It is calculated using diagnoses for 17 diseases abstracted from hospital data. When present, each condition is assigned a score from one to six and the scores are summed to give a single value ranging from 0 to 32, where a higher score indicates a greater burden of comorbidity. It was originally created using ICD-9 codes but has been verified using ICD-10-CA codes [28,29] as well as physician data [30]. We used diagnoses codes from both hospital and physician data to calculate the Charlson index using Quan et al's (2006) version [31,32]. For the hospital data, diagnoses that developed after hospital admission and which represent complications of the hospitalization were excluded.

The Elixhauser index identifies the presence of 31 diseases using administrative data [5]. Each condition is coded as present or absent and is entered into a statistical model as its own variable. Similar to the Charlson index, the Elixhauser index was originally created using ICD-9 codes but has since been verified using ICD-10-CA codes [29]. We used diagnoses codes from both hospital and physician data to calculate the Elixhauser index using Quan et al's (2006) version [33,34]. For the hospital data, diagnoses that developed after hospital admission and which represent complications of the hospitalization were excluded.

The number of dispensed drugs was calculated using the American Hospital Formulary Service (AHFS) pharmacologic-therapeutic classification system by summing the number of different four-digit drug classifications for each cohort member to a maximum of 125. The Chronic Disease Score (CDS) is also calculated based upon the AHFS classification system [3]. Drugs used to treat 17 conditions are identified and assigned a score from one to five. The scores are summed to give a single value ranging from 0 to 35, where a high score indicates a greater burden of comorbidity. The CDS predicts both hospitalization and mortality and is positively correlated with physician ratings of disease severity (r = 0.57) [3].

### Outcome variables

Study outcomes included death and hospitalization between April 1, 2002 and March 31, 2003. Death was determined using data from the population registry, vital statistics, and hospitalization databases. Two measures of hospital use were examined: at least one hospitalization and two or more hospitalizations. Hospitalizations related to pregnancy, childbirth, and abortion were excluded from the analysis. We investigated multiple hospitalizations because hospitalization was a relatively frequent occurrence in the chronic disease cohorts.

### Other study variables

Other variables used to describe the cohorts include age, sex, region of residence, and income quintile. All demographic variables were determined for fiscal year 2001/02. Age and sex were identified from the Population Registry. Region of residence and income were both determined using a resident's postal code identified from the *Population Registry*. Income is a well known factor that influences health outcomes, such that lower income individuals are more likely to become ill, disabled, and die than their more affluent counterparts [35]. Region of residence may also affect health and the use of health services. Nearly half of Saskatchewan residents live in a rural area and may face barriers to accessing health care services that urban residents do not. A resident was categorized as living in an urban area if his/her postal code was in a Census Metropolitan Area or Census Agglomeration with a population of 10,000 or more.

Income quintiles were calculated using a method based on average household income from the 2001 Statistics Canada Census [36]. Each resident's postal code was identified from the *Population Registry *and linked to a dissemination area, the smallest geographic unit used in Census data. Residents were identified as belonging to an income quintile based upon the *Income Per Person Equivalent *which takes the size of a household into consideration. Income quintiles were calculated so that the entire population of Saskatchewan was divided into five equal groups. Some residents could not be assigned to a quintile because income measures are suppressed for DAs with a small population. Approximately 14% of the total Saskatchewan population had a missing income quintile. Imputed values were assigned using a method that creates a predictive model for the missing quintiles using sociodemographic variables that are not suppressed such as marital status, ethnicity, and employment status. A multiple imputation approach was then used to assign income quintile [37]. After applying this methodology, income could still not be assigned to some rural areas in which approximately one percent of Saskatchewan residents lived in fiscal year 2001/02.

### Statistical analysis

In order to ensure comparability with the general population, we restricted both chronic disease cohorts to individuals who were alive on April 1, 2002, and who had uninterrupted health coverage for fiscal years 2001/02 and 2002/03. Two sets of analyses were conducted; one including all members of each cohort and the other including only those cohort members age 65 and older.

Frequencies, means, and standard deviations were used to describe the characteristics of each cohort. The chronic disease cohorts are subsets of the general population cohort and so McNemar's test was used to test for differences between the cohorts on each of the three outcome measures. Validation of each comorbidity measure was conducted using multiple logistic regression analysis [15]. Specifically, to assess the predictive performance of each comorbidity measure a series of models were fit to the data for each outcome. The base model was comprised of the following variables: age (in years), a quadratic age effect, sex, region of residence, and income quintile. Five full models were then fit to the data. Each model contained all of the variables in the base model in addition to one or more variables defining the comorbidity measure. All comorbidity measures were entered into the full models as continuous variables with the exception of the Elixhauser index, which was included as a series of dichotomous variables. Sensitivity analyses revealed that redefining the continuous comorbidity measures as categorical variables did not result in any substantial change in model fit as judged by the Hosmer-Lemeshow goodness-of-fit test.

Discriminative performance for the base and full models was assessed using the c-statistic, which is equivalent to the area under the receiver operating characteristic (ROC) curve for dichotomous outcomes [27,38]. The c-statistic ranges from zero to one, with a value of one representing perfect prediction and a value of 0.5 representing chance prediction. A value between 0.7 and 0.8 is considered to demonstrate acceptable predictive performance, while a value greater than 0.8 demonstrates excellent discriminative performance. The 95% confidence intervals (CIs) were computed. Differences in the c-statistic (i.e., Δc) for the base and full models were tested using the method of DeLong et al. [39]. The percentage change in the c-statistic was also computed.

Model calibration was assessed using the Brier score, which ranges from zero to one [28]. A lower score indicates less prediction error. Given that a score of 0.25 can be achieved by assigning an event probability of 0.5 to each individual [28], a value less than 0.25 was considered to represent acceptable prediction error. The standard deviation of the Brier score was also computed.

Analyses were conducted for each full cohort and then for the age-restricted (65+) cohort using SAS software [40].

## Results

Table 1 contains summary data for the demographic and socioeconomic variables, health outcomes, and comorbidity measures for each cohort. We describe first the findings for the full cohorts. We identified 662,423 individuals in the general population cohort, 41,925 individuals age in the diabetes cohort, and 28,028 individuals in the osteoporosis cohort. A total of 2,909 individuals were members of both the diabetes cohort (6.9%) and osteoporosis cohort (10.4%). The general population cohort was, on average, younger than the diabetes cohort [mean age (SD) 47.9(17.9) versus 62.4(15.0)]. Females represented approximately 50% of both the general population and diabetes cohort and the majority of the osteoporosis cohort (86.1%). A greater proportion of members of the diabetes cohort had incomes in the lowest quintile (26.1%) than both the general population and osteoporosis cohorts. The majority of individuals lived in an urban setting, ranging from 52% in the diabetes cohort to 58% in both the general population and osteoporosis cohort.

**Table 1:** Description of general population, diabetes, and osteoporosis full and age-restricted cohorts

	General Population		Diabetes		Osteoporosis	
**Variable**	**Full (*n *= 662,423)**	**65+ years (*n *= 137,700)**	**Full (*n *= 41,925)**	**65+ years (*n *= 20,025)**	**Full (*n *= 28,068)**	**65+ years** **(*n *= 20,090)**

**DEMOGRAPHICS**						

Age, mean (SD)	47.9 (17.9)	75.3 (7.3)	62.4 (15.0)	75.2 (6.9)	71.6 (10.8)	77.0 (7.4)

Female, %	51.3	56.8	47.8	49.7	86.1	87.1

Urban residence, %	58.2	51.1	51.8	49.5	58.0	56.8

Missing	0.2	0.1	0.1	0.1	0.1	0.1

Income quintile, %						

Q1 (lowest)	21.6	22.2	26.1	24.7	22.2	23.3

Q2	22.0	23.6	23.0	23.6	22.3	22.7

Q3	18.2	18.7	17.0	18.3	18.7	19.2

Q4	16.6	15.8	14.8	15.0	17.0	16.5

Q5 (highest)	20.4	18.4	17.9	17.2	18.9	17.5

Missing	1.2	1.3	1.2	1.3	1.0	0.8

**OUTCOMES**						

Death, %	1.3	5.1	4.3	7.5	4.7	6.1

One or more hospitalizations, %	17.4	31.8	31.9	39.9	33.9	37.2

Two or more hospitalizations, %	5.1	12.6	13.2	17.9	14.8	16.9

**COMORBIDITY MEASURES**						

# Diagnoses, mean (SD)	3.9 (4.4)	6.3 (5.6)	7.5 (6.4)	8.4 (6.7)	8.0 (6.2)	8.5 (6.4)

Charlson index summary score, mean (SD)	0.3 (0.9)	0.7 (1.5)	0.8 (1.5)	1.1 (1.8)	0.8 (1.5)	0.9 (1.5)

# Drugs, mean (SD)	1.8 (1.0)	2.5 (1.2)	5.1 (3.9)	6.1 (3.9)	4.1 (2.4)	4.4 (2.4)

CDS, mean (SD)	1.4 (2.6)	3.3 (3.4)	4.8 (3.8)	5.6 (3.7)	3.6 (3.6)	4.0 (3.6)

All three outcomes occurred less frequently in the general population cohort than in either of the other two cohorts. Each outcome was two to three times more common in the diabetes and osteoporosis cohorts. These differences were statistically significant for both hospitalization outcomes but not for death.

For each comorbidity measure, the general population cohort had the lowest mean score. The osteoporosis cohort had the highest mean number of diagnoses while the diabetes cohort had the highest mean number of drugs and chronic disease score. Mean Charlson index summary scores were identical in the osteoporosis and diabetes cohorts. The Charlson value is less than one in these disease-based cohorts because the cohorts were created using diagnosis codes recorded over a 6-year period, whereas the comorbidity measures were created using diagnosis codes recorded within a single year.

Table 2 summarizes the distribution of Elixhauser categories. In each full cohort, uncomplicated hypertension was the most common chronic condition followed by chronic pulmonary disease. Few members of the general population had congestive heart failure (2.0%) although it was the third and fourth most common chronic condition in the diabetes cohort (8.0%) and osteoporosis cohort (8.2%), respectively. The prevalence of each chronic condition was lowest in the general population cohort for all Elixhauser categories. Compared to the other cohorts, members of the diabetes cohort were more likely to be identified as having complicated diabetes (14.7%) and renal failure (3.1%), while members of the osteoporosis cohort were more likely to be identified as having hypothyroidism (9.0%), depression (7.5%), and rheumatic disease (6.9%).

**Table 2:** Elixhauser Index categories for the general population, diabetes, and osteoporosis full and age-restricted cohorts, 2001/02

	General Population		Diabetes		Osteoporosis	
**Variable**	**Full** **(%)**	**65+ years** **(%)**	**Full** **(%)**	**65+ years** **(%)**	**Full** **(%)**	**65+ years** **(%)**

Hypertension, uncomplicated	16.7	42.5	42.7	51.1	39.9	44.8

Chronic pulmonary disease	8.4	12.7	13.5	14.3	15.2	15.9

Depression	5.6	4.6	6.0	4.8	7.5	7.1

Hypothyroidism	3.4	6.6	4.7	5.6	9.0	9.4

Solid tumor	2.5	7.6	5.4	8.8	6.7	7.3

Congestive heart failure	2.0	8.1	8.0	13.4	8.2	10.7

Psychiatric disorder	1.3	4.1	2.7	4.4	4.3	5.6

Rheumatic disease	1.2	2.4	1.9	2.2	6.9	7.0

Diabetes, complicated	1.0	2.8	14.7	18.5	2.3	2.6

Valvular disease	1.0	2.6	2.2	3.1	2.8	3.3

Other neurological disorders	1.0	1.3	1.2	1.3	1.8	1.7

Cardiac arrhythmias	0.8	3.2	2.8	4.7	3.0	3.9

Fluid and electrolyte disorders	0.8	2.5	2.7	4.0	3.2	4.1

Coagulopathies	0.8	2.8	2.5	3.9	2.9	3.6

Metastatic cancer	0.8	2.4	1.7	2.8	2.4	2.6

Renal failure	0.6	1.8	3.1	4.2	1.9	2.3

Drug abuse	0.5	0.1	0.4	0.1	0.2	0.1

Peripheral vascular disease	0.4	1.3	1.5	2.2	1.2	1.5

Deficiency anemia	0.4	1.0	0.8	1.3	1.3	1.5

Hypertension, complicated	0.3	0.9	1.0	1.5	1.0	1.2

Pulmonary circulation disorders	0.3	1.0	0.8	1.2	1.2	1.4

Liver disease	0.3	0.3	0.7	0.6	0.5	0.4

Alcohol abuse	0.3	0.2	0.7	0.4	0.2	0.2

Diabetes, uncomplicated	0.2	0.5	3.0	3.3	0.4	0.4

Obesity	0.2	0.3	1.0	0.9	0.3	0.3

Paraplegia	0.2	0.3	0.4	0.6	0.3	0.3

Peptic ulcer disease	0.1	0.4	0.4	0.5	0.4	0.5

Lymphoma	0.1	0.2	0.2	0.3	0.3	0.3

Weight loss	0.1	0.2	0.2	0.2	0.3	0.3

Blood loss anemia	<0.1	0.1	0.1	0.1	0.1	0.1

AIDS	<0.1	<0.1	<0.1	0	0	0

When we restricted attention to only those cohort members who were 65+ years, we found the general population and diabetes cohorts were similar in terms of age and urban residence, while the osteoporosis cohort was slightly older and composed of more women (Table 1). While mean scores for all comorbidity measures increased, the pattern across the age-restricted cohorts was similar to that for the full cohorts.

Table 3 reports the results of the multivariable analyses for the full and age-restricted general population cohorts. The base model for death in the full cohort had a c-statistic of 0.880 (95% CI: 0.886, 0.892) and a Brier score of 0.012, indicating excellent discrimination and very low prediction error. The addition of a comorbidity measure to the base model yielded a statistically significant improvement in the c-statistic for all five measures, although the amount of change was small (< 4%). The addition of the Elixhauser index to the base model was associated with the largest c-statistic (c = 0.913; 95% CI: 0.910, 0.916), followed by the Charlson index (c = 0.905; 95% CI: 0.902, 0.908). Hospitalization base models had markedly lower c-statistics than the mortality base model. In fact, the c-statistic for the base model for any hospitalization in the full cohort failed to exceed the threshold of 0.70 and barely surpassed it for multiple hospitalizations (c = 0.706; 95% CI: 0.704, 0.709). For each model, the addition of every comorbidity measure was associated with a statistically significant increase in the c-statistic. The number of diagnoses was the best-performing comorbidity measure for the models of one or more hospitalizations (c = 0.722; 95% CI: 0.720, 0.724) and two or more hospitalizations (c = 0.782; 95% CI: 0.779, 0.785).

**Table 3:** Model comparisons for mortality and hospitalization in the general population cohort, full and age-restricted

Model	Full cohort (n = 662,423)			Age 65+ (n = 137,700)		
	***c*-statistic** **(95% CI)**	**Brier** **score (SD)**	**Δ*c *(%)**	***c*-statistic** **(95% CI)**	**Brier** **score (SD)**	**Δ *c *(%)**

**Death**						

Base model	0.880 (0.877, 0.884)	0.012 (0.098)	--	0.729 (0.723, 0.735)	0.046 (0.183)	--

+ # diagnoses	**0.901** (0.898, 0.904)	0.012 (0.096)	0.021 (2.36)	**0.769** (0.764, 0.775)	0.046 (0.179)	0.040 (5.53)

+ Charlson	**0.905** (0.902, 0.908)	0.012 (0.095)	0.025 (2.81)	**0.785** (0.779, 0.790)	0.045 (0.177)	0.056 (7.64)

+ Elixhauser	**0.913** (0.910, 0.916)	0.012 (0.093)	0.033 (3.73)	**0.805** (0.799, 0.810)	0.044 (0.173)	0.076 (10.36)

+ # drugs	**0.894** (0.890, 0.897)	0.012 (0.096)	0.013 (1.53)	**0.764** (0.759, 0.770)	0.045 (0.178)	0.035 (4.83)

+ CDS	**0.889** (0.886, 0.892)	0.012 (0.097)	0.009 (1.01)	**0.751** (0.745, 0.757)	0.046 (0.181)	0.022 (3.00)

**One or more hospitalizations**						

Base model	0.652 (0.651, 0.654)	0.138 (0.238)	--	0.563 (0.559, 0.566)	0.215 (0.171)	--

+ # diagnoses	**0.722** (0.720, 0.724)	0.130 (0.233)	0.070 (10.68)	**0.668** (0.664, 0.671)	0.202 (0.189)	0.105 (18.60)

+ Charlson	**0.671** (0.669, 0.672)	0.136 (0.238)	0.018 (2.81)	**0.613** (0.610, 0.616)	0.210 (0.179)	0.050 (8.92)

+ Elixhauser	**0.682** (0.680, 0.683)	0.134 (0.236)	0.029 (4.47)	**0.630** (0.627, 0.633)	0.206 (0.184)	0.067 (11.92)

+ # drugs	**0.688** (0.686, 0.690)	0.134 (0.235)	0.036 (5.46)	**0.625** (0.622, 0.628)	0.207 (0.181)	0.063 (11.10)

+ CDS	**0.672** (0.671, 0.674)	0.136 (0.236)	0.020 (3.05)	**0.604** (0.601, 0.607)	0.210 (0.177)	0.041 (7.34)

**Two or more hospitalizations**						

Base model	0.706 (0.704, 0.709)	0.047 (0.187)	--	0.571 (0.567, 0.576)	0.110 (0.246)	--

+ # diagnoses	**0.782** (0.779, 0.785)	0.045 (0.179)	0.075 (10.67)	**0.686** (0.682, 0.690)	0.105 (0.235)	0.115 (20.07)

+ Charlson	**0.731** (0.728, 0.734)	0.047 (0.184)	0.024 (3.42)	**0.633** (0.629, 0.638)	0.108 (0.241)	0.062 (10.92)

+ Elixhauser	**0.748** (0.745, 0.751)	0.046 (0.181)	0.042 (5.91)	**0.653** (0.649, 0.658)	0.106 (0.237)	0.082 (14.40)

+ # drugs	**0.744** (0.742, 0.747)	0.046 (0.182)	0.038 (5.37)	**0.638** (0.633, 0.642)	0.107 (0.238)	0.067 (11.66)

+ CDS	**0.729** (0.726, 0.732)	0.047 (0.184)	0.023 (3.18)	**0.619** (0.614, 0.623)	0.108 (0.241)	0.048 (8.32)

Base model includes age, age^2^, sex, income quintile, and geography

CDS = Chronic Disease Score

Δ*c *= difference in the *c*-statistic between the base and full models; *c*-statistics in boldface font are significantly different from the *c*-statistic for the base model, according to the method of DeLong et al. [39]

Table 4 provides the multivariable models for the full and age-restricted diabetes cohorts. Similar to the general population cohort, the base model for death had the largest c-statistic in the full cohort (c = 0.781; 95% CI: 0.770, 0.791). The addition of each comorbidity measure to the base model resulted in a significant improvement in the c-statistic. The greatest improvement was observed for the Elixhauser index (c = 0.845; 95% CI: 0.836, 0.854), followed by the Charlson index (c = 0.830; 95% CI: 0.821, 0.839). C-statistics for the base models predicting hospitalization were below 0.70, and although the addition of each comorbidity measure resulted in a statistically significant improvement in the c-statistic, few models exceeded the threshold of 0.70. The number of diagnoses was associated with largest c-statistic for at least one hospitalization (c- = 0.701; 95%CI: 0.695, 0.672) and two or more hospitalizations (c = 0.726; 95%CI: 0.719, 0.733). Overall, there was better predictive performance in the general cohort than in the diabetes cohort for all outcomes.

**Table 4:** Model comparisons for mortality and hospitalization for diabetes cohort, full and age-restricted

Model	Full cohort (n = 41,925)			Age 65+ (n = 20,025)		
	***c*-statistic** **(95% CI)**	**Brier** **score (SD)**	**Δ *c *(%)**	***c*-statistic** **(95% CI)**	**Brier** **score (SD)**	**Δ *c *(%)**
**Death**						

Base model	0.781 (0.770, 0.791)	0.038 (0.166)	--	0.695 (0.680, 0.709)	0.067 (0.209)	--

+ # diagnoses	**0.818** (0.809, 0.828)	0.037 (0.161)	0.037 (4.78)	**0.749** (0.736, 0.762)	0.065 (0.202)	0.054 (7.81)

+ Charlson	**0.830** (0.821, 0.839)	0.037 (0.161)	0.049 (6.33)	**0.764** (0.752, 0.776)	0.065 (0.202)	0.070 (10.02)

+ Elixhauser	**0.845** (0.836, 0.854)	0.036 (0.156)	0.064 (8.20)	**0.788** (0.776, 0.800)	0.063 (0.196)	0.094 (13.50)

+ # drugs	**0.799** (0.789, 0.810)	0.038 (0.163)	0.018 (2.36)	**0.729** (0.715, 0.742)	0.065 (0.204)	0.034 (4.92)

+ CDS	**0.789** (0.779, 0.800)	0.038 (0.165)	0.009 (1.11)	**0.709** (0.695, 0.723)	0.066 (0.207)	0.014 (2.07)

**One or more hospitalizations**						

Base model	0.610 (0.604, 0.616)	0.211 (0.174)	--	0.547 (0.539, 0.556)	0.238 (0.104)	--

+ # diagnoses	**0.701** (0.695, 0.706)	0.195 (0.194)	0.091 (14.90)	**0.655** (0.647, 0.663)	0.224 (0.148)	0.108 (19.63)

+ Charlson	**0.666** (0.660, 0.672)	0.203 (0.187)	0.056 (9.21)	**0.621** (0.613, 0.629)	0.231 (0.130)	0.074 (13.45)

+ Elixhauser	**0.677** (0.671, 0.682)	0.198 (0.192)	0.067 (10.98)	**0.634** (0.626, 0.642)	0.226 (0.143)	0.086 (15.76)

+ # drugs	**0.641** (0.635, 0.647)	0.206 (0.181)	0.031 (5.11)	**0.604** (0.596, 0.612)	0.232 (0.125)	0.056 (10.28)

+ CDS	**0.624** (0.618, 0.630)	0.208 (0.177)	0.014 (2.31)	**0.578** (0.570, 0.587)	0.235 (0.115)	0.031 (5.65)

**Two or more hospitalizations**						

Base model	0.617 (0.609, 0.625)	0.113 (0.242)	--	0.546 (0.535, 0.556)	0.146 (0.245)	--

+ # diagnoses	**0.726** (0.719, 0.733)	0.106 (0.230)	0.109 (17.61)	**0.674** (0.665, 0.684)	0.139 (0.237)	0.129 (23.55)

+ Charlson	**0.694** (0.687, 0.702)	0.110 (0.236)	0.077 (12.55)	**0.643** (0.633, 0.653)	0.143 (0.240)	0.097 (17.76)

+ Elixhauser	**0.709** (0.702, 0.717)	0.107 (0.232)	0.092 (14.96)	**0.655** (0.645, 0.665)	0.139 (0.237)	0.109 (20.02)

+ # drugs	**0.651** (0.643, 0.658)	0.111 (0.237)	0.034 (5.45)	**0.603** (0.592, 0.614)	0.144 (0.241)	0.057 (10.49)

+ CDS	**0.633** (0.625, 0.641)	0.112 (0.240)	0.016 (2.63)	**0.580 **(0.569, 0.590)	0.145 (0.242)	0.034 (6.22)

Base model includes age, age^2^, sex, income quintile, and geography

CDS = Chronic Disease Score

Δ*c *= difference in the *c*-statistic between the base and full models; *c*-statistics in boldface font are significantly different from the *c*-statistic for the base model, according to the method of DeLong et al. [39]

Table 5 reports the results of the multivariable models for the full and age-restricted osteoporosis cohorts. The base model for death had the largest c-statistic (c = 0.758; 95% CI: 0.745, 0.771). The addition of each comorbidity measure to the base model was associated with a statistically significant improvement in the c-statistic, although the amount of change was largest for the Elixhauser index (c = 0.827: 95% CI: 0.817, 0.838), followed by the Charlson index (c = 0.811; 95% CI: 0.800, 0.822). For both hospitalization outcomes, the c-statistic of all models failed to exceed 0.70. The number of diagnoses was associated with the greatest improvement in the c-statistic for at least one hospitalization (c-= 0.667; 95% CI: 0.660, 0.673) as well as for two or more hospitalizations (c = 0.688; 95% CI: 0.688, 0.705). Overall, there was better predictive performance in the general cohort than in the diabetes cohort for all outcomes.

**Table 5:** Model comparisons for mortality and hospitalization for osteoporosis cohort, full and age-restricted

Model	Full cohort (n = 28,068)			Age 65+ (n = 20,090)		
	***c*-statistic** **(95% CI)**	**Brier** **score (SD)**	**Δ *c *(%)**	***c*-statistic** **(95% CI)**	**Brier** **Score (SD)**	**Δ *c *(%)**

**Death**						

Base model	0.758 (0.745, 0.771)	0.042 (0.175)	--	0.718 (0.703, 0.733)	0.055 (0.196)	--
+ # diagnoses	**0.792** (0.780, 0.804)	0.042 (0.171)	0.034 (4.48)	**0.756** (0.742, 0.769)	0.054 (0.191)	0.038 (5.29)

+ Charlson	**0.811** (0.800, 0.822)	0.042 (0.170)	0.053 (6.95)	**0.772** (0.759, 0.785)	0.054 (0.190)	0.054 (7.57)

+ Elixhauser	**0.827** (0.817, 0.838)	0.040 (0.166)	0.069 (9.09)	**0.793** (0.780, 0.805)	0.052 (0.185)	0.075 (10.42)

+ # drugs	**0.782** (0.769, 0.794)	0.042 (0.172)	0.024 (3.10)	**0.746** (0.732, 0.760)	0.054 (0.191)	0.028 (3.91)

+ CDS	**0.778** (0.766, 0.791)	0.042 (0.172)	0.020 (2.62)	**0.741** (0.727, 0.754)	0.054 (0.192)	0.023 (3.15)

**One or more hospitalizations**						

Base model	0.579 (0.572, 0.586)	0.220 (0.157)	--	0.550 (0.542, 0.558)	0.232 (0.129)	--
+ # diagnoses	**0.667** (0.660, 0.673)	0.208 (0.178)	0.088 (15.14)	**0.647** (0.639, 0.654)	0.220 (0.157)	0.097 (17.54)

+ Charlson	**0.619** (0.612, 0.626)	0.216 (0.166)	0.040 (6.98)	**0.598** (0.590, 0.606)	0.227 (0.141)	0.048 (8.73)

+ Elixhauser	**0.637** (0.630, 0.644)	0.212 (0.173)	0.058 (10.08)	**0.620** (0.612, 0.628)	0.223 (0.151)	0.070 (12.65)

+ # drugs	**0.631** (0.624, 0.638)	0.213 (0.168)	0.052 (9.02)	**0.609** (0.601, 0.618)	0.225 (0.144)	0.059 (10.80)

+ CDS	**0.618** (0.611, 0.625)	0.215 (0.166)	0.039 (6.69)	**0.597** (0.589, 0.605)	0.227 (0.141)	0.047 (8.61)

**Two or more hospitalizations**						

Base model	0.606 (0.597, 0.615)	0.124 (0.243)	--	0.570 (0.559, 0.580)	0.139 (0.245)	--

+ # diagnoses	**0.697** (0.688, 0.705)	0.118 (0.235)	0.090 (14.92)	**0.669** (0.659, 0.679)	0.133 (0.238)	0.099 (17.37)

+ Charlson	**0.650** (0.641, 0.659)	0.122 (0.240)	0.044 (7.19)	**0.619** (0.609, 0.630)	0.137 (0.242)	0.050 (8.73)

+ Elixhauser	**0.672** (0.663, 0.681)	0.119 (0.237)	0.066 (10.86)	**0.645** (0.634, 0.655)	0.135 (0.239)	0.075 (13.15)

+ # drugs	**0.656** (0.647, 0.665)	0.121 (0.237)	0.050 (8.28)	**0.625** (0.615, 0.636)	0.136 (0.240)	0.056 (9.80)

+ CDS	**0.651** (0.642, 0.660)	0.121 (0.238)	0.045 (7.41)	**0.620** (0.610, 0.631)	0.137 (0.240)	0.051 (8.89)

Base model includes age, age^2^, sex, income quintile, and geography

CDS = Chronic Disease Score

Δ*c *= difference in the *c*-statistic between the base and full models; *c*-statistics in boldface font are significantly different from the *c*-statistic for the base model, according to the method of DeLong et al. [39]

Results for the age-restricted cohorts were similar to the results for each full cohort. C-statistics were lower and Brier scores were higher in all models in the age-restricted cohort compared to their equivalent models in full cohort, but the same comorbidity measures were identified as having the greatest improvement in predictive performance. Additionally, the change in the c-statistic was greater for each model in the age-restricted cohort model than for the counterpart model in the full cohort.

## Discussion

This research comparing discrimination and prediction error associated with comorbidity measures in three population-based cohorts has the following key findings. First, the optimal comorbidity measure depends upon the outcome of interest. However, for each of the outcomes, the best performing comorbidity measures was consistent across populations with different disease characteristics. Finally, compared to the full general population cohort, models in the chronic disease and age-restricted cohorts had poorer discriminative performance of a base set of demographic and socioeconomic variables, but the improvement in discriminative performance associated with a comorbidity measure was consistently larger.

We found that the best performing comorbidity measure depends upon the outcome of interest. The number of different diagnoses in both hospital and physician databases was the best predictor of hospitalization, whereas the best predictors of death were the Elixhauser and Charlson indices. Our findings for health services utilization are similar to those of Farley et al., who found that number of diagnoses and number of drugs performed better than the Elixhauser and Charlson indices for predicting health care expenditures in a general population [41]. However, Perkins et al. reported that a simple count of medications was the best predictor of health care costs in a population-based cohort of community-dwelling older adults aged 60 and older [7]. Perkins et al. investigated many of the same comorbidity measures that we did including count of diagnoses, count of drugs, CDS, and the Charlson index using both hospital and physician data. The discrepancy between Perkins' and our findings may be the result of differences in how the number of drugs was ascertained. Perkins et al. had a pharmacist divide drugs into detailed subclasses whereas we used the AHFS to the fourth digit, which is not as specific. Another possible explanation is that although Perkins et al. included a count of diagnoses as one of their comorbidity measures, the researchers restricted the count to ten common chronic conditions, whereas we identified all conditions based on diagnoses codes recorded in hospital and physician databases.

Our finding that the Elixhauser and Charlson indices are the optimal predictors of one-year mortality is also in keeping with other studies of different populations including in-patient populations, community-dwelling older adults, and hypertensive adults [7,9,12,42]. Furthermore, studies that directly compare the Elixhauser to Charlson indices found, as we did, that the Elixhauser index performed better than the Charlson index [6,43,44].

Our findings show that, although the optimal comorbidity measure varies by outcome, results are remarkably consistent across study populations with different disease profiles. This consistency in the performance of comorbidity measures predicting one-year mortality was also observed by Stukenborg et al. (2001) in five in-patient populations (acute myocardial infarction, congestive heart failure, chronic obstructive pulmonary disease, hypertension with complications, and acute cerebrovascular disease), although they limited their investigation to only the Elixhauser and Charlson indices. Similarly, in 2004 Schneeweiss et al. compared the discriminative performance of four versions of the Charlson index and two versions of the CDS for predicting one-year mortality. They found the rank order for the best performing comorbidity measures was identical in four cohorts of community-dwelling adults age 64 and older - two with cardiovascular disease and two without restrictions. Our results confirm these findings, but further expand upon their work by investigating additional comorbidity measures, investigating health services utilization as an outcome, and including adults of all ages in the study cohorts.

Although the rank ordering of comorbidity measures in terms of discriminant performance was consistent across our study populations, we found that performance was poorer in the chronic disease cohorts. Compared to identical models in the general population cohort, models in the diabetes and osteoporosis cohorts had poorer discriminative performance (i.e., lower c-statistic values) and higher prediction error (i.e., higher Brier scores). This may be the result of not including important disease-specific variables in our models such as the recency of diagnoses or exposure to certain medications. We did not include them in order to make the models directly comparable between study cohorts. Particularly for health services outcomes, for which the c-statistic rarely surpassed the cutoff of 0.70, adding other variables might improve the performance of these models.

We also observed diminished performance in the age-restricted cohorts compared to the full cohorts although the magnitudes of change in the c-statistics were consistently larger. The increase in the magnitudes of change in the c-statistics may be explained by the fact that advancing age is associated with the presence of multiple chronic illnesses [45]. The high prevalence of chronic disease would account for a greater proportion of the variation observed in the age-restricted cohorts compared to the full cohort and which would correspond to a proportionately greater increase in the c-statistic.

The strengths of this study include the use of population-based administrative databases and our comprehensive investigation of multiple comorbidity measures with multiple outcomes in a variety of study populations. Furthermore, the cohorts are population-based which ensures good generalizability of our findings.

There are some limitations to our research. Misclassification bias for specific comorbid conditions may occur as a result of inaccurate diagnosis coding in the administrative databases. Specifically, physicians may code a suspected diagnosis only to rule it out later and which would falsely inflate scores for diagnosis-based comorbidity measures. As well, comorbidity measures were constructed using a single year of administrative data so individuals that have a disease but did not have any health service records containing a diagnosis of that disease during the one year timeframe would not be captured in the comorbidity measure for that disease. For example, of the diabetes cohort identified for this study - based on diagnosis codes occurring during a 6-year period - only 15% had a diabetes diagnosis code occurring during the one-year period upon which the Elixhauser index was based. We could have reduced the likelihood for misclassification of the comorbidity measures by expanding the time-frame for ascertainment of comorbidity to more than one year, but a one-year time frame has been adopted in other studies [7-9] and enables direct comparison with our results. Furthermore, a study comparing varying time frames for the assessment of comorbidity in the prediction of one-year mortality found that a longer time frame did not improve the c-statistic substantially [46]. Another limitation is that our finding that the best performing comorbidity measure is consistent across cohorts may be because the chronic disease cohorts are subsets of the general population cohort. This is unlikely though, given that people with diabetes and/or osteoporosis comprise only about 5% of the full general population cohort and 15% of the age 65+ general population cohort.

## Conclusions

Based on the results of this research, the optimal comorbidity measure is primarily dependent upon the outcome of interest and not on the study population. Overall, the count of diagnoses had the best predictive performance for hospital utilization while the Elixhauser index, followed by the Charlson index, had the best predictive performance for mortality. These findings were consistent in a general population cohort and two chronic disease cohorts, even when analyses were restricted to older adults.

## Competing interests

JQ: No disclosures

LL: Unrestricted research grant from Amgen Canada

GT: No disclosures

BA: No disclosures

## Authors' contributions

JQ developed the study concept, analyzed the data, interpreted results, and prepared the manuscript. LL developed the study concept and methodology, and interpreted results. BA assisted with the literature review and data analysis. GT developed the study concept. All authors read and approved the final manuscript.

## Pre-publication history

The pre-publication history for this paper can be accessed here:

http://www.biomedcentral.com/1472-6963/11/146/prepub
